# Supplementary material for: DMRforPairs: identifying Differentially Methylated Regions between unique samples using array based methylation profiles
Source: BMC Bioinformatics. 2014 May 15;15:141. doi: 10.1186/1471-2105-15-141 (PMC4046028; doi:10.1186/1471-2105-15-141)
Supplement: Additional file 2 — DMRforPairs output for the comparison of A431-MCF7 and NA17018-NA17105. Please start from the HTML files in each folder. Available via the BMC Bioinformatics website. [file 1471-2105-15-141-S2.zip › 1394847754114233_MOESM2_ESM/A431_MCF7/figures/16894.pdf]

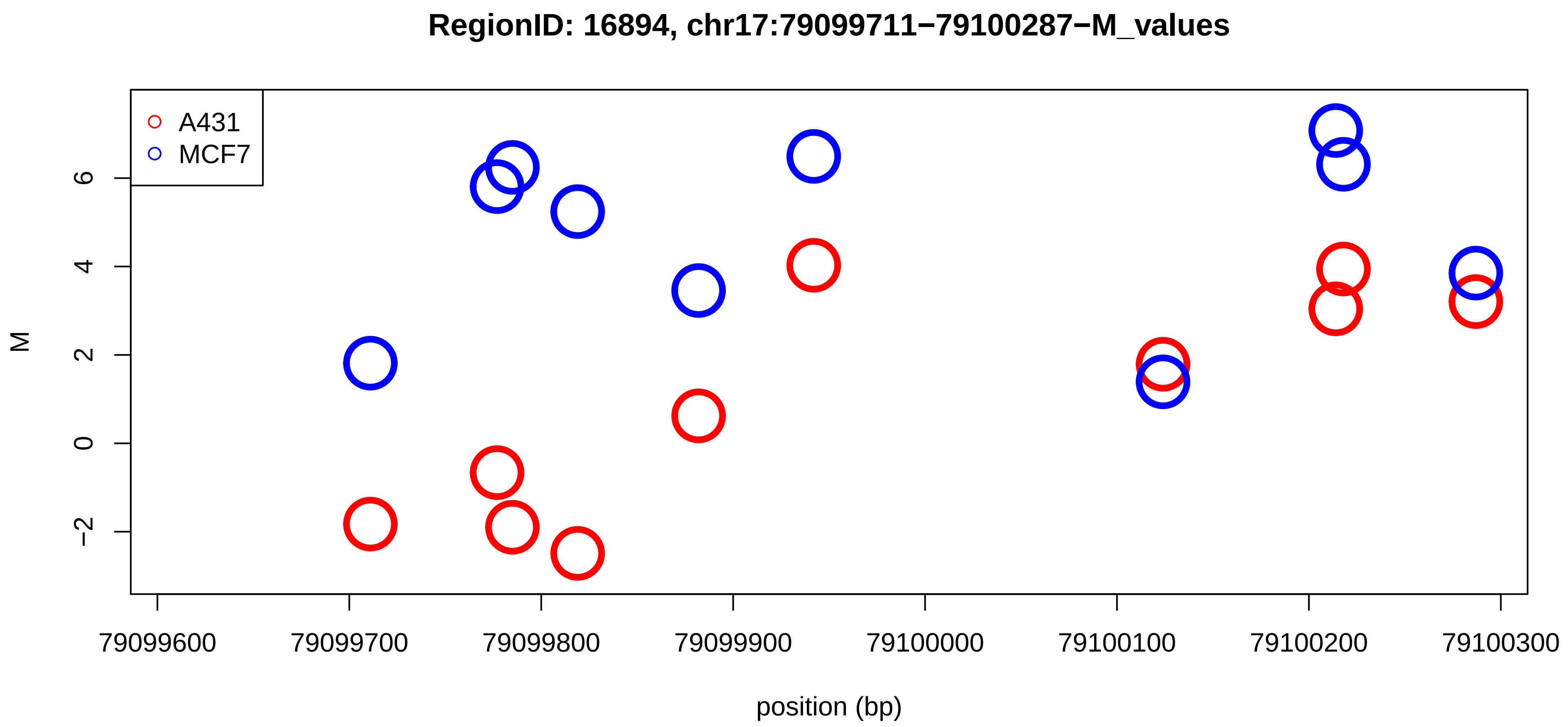
RegionID: 16894, chr17:79099711−79100287−M_values
A431
MCF7
M
−2
0
2
4
6
79099600
79099700
79099800
79099900
79100000
79100100
79100200
79100300
position (bp)

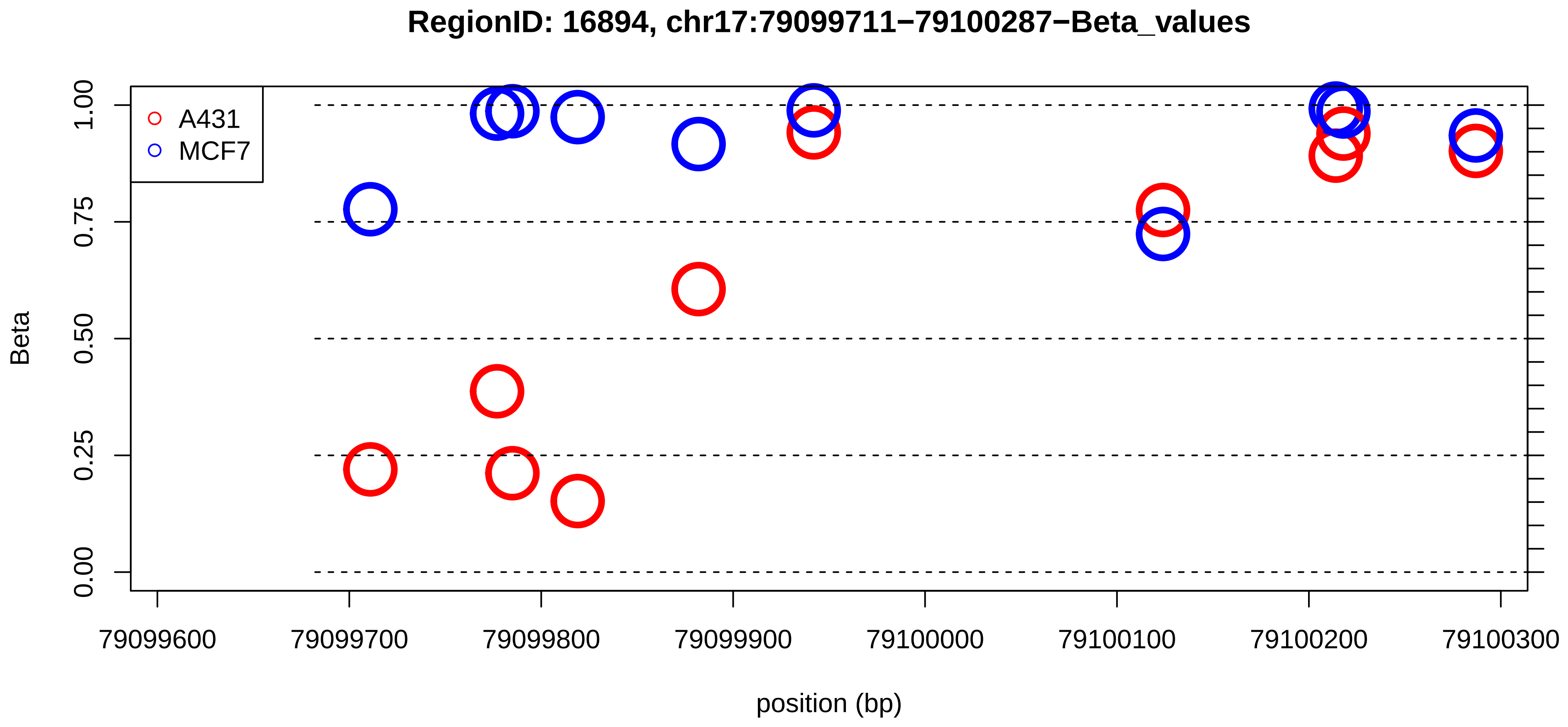
RegionID: 16894, chr17:79099711−79100287−Beta_values
A431
MCF7
Beta
0.00
0.25
0.50
0.75
1.00
79099600
79099700
79099800
79099900
79100000
79100100
79100200
79100300
position (bp)

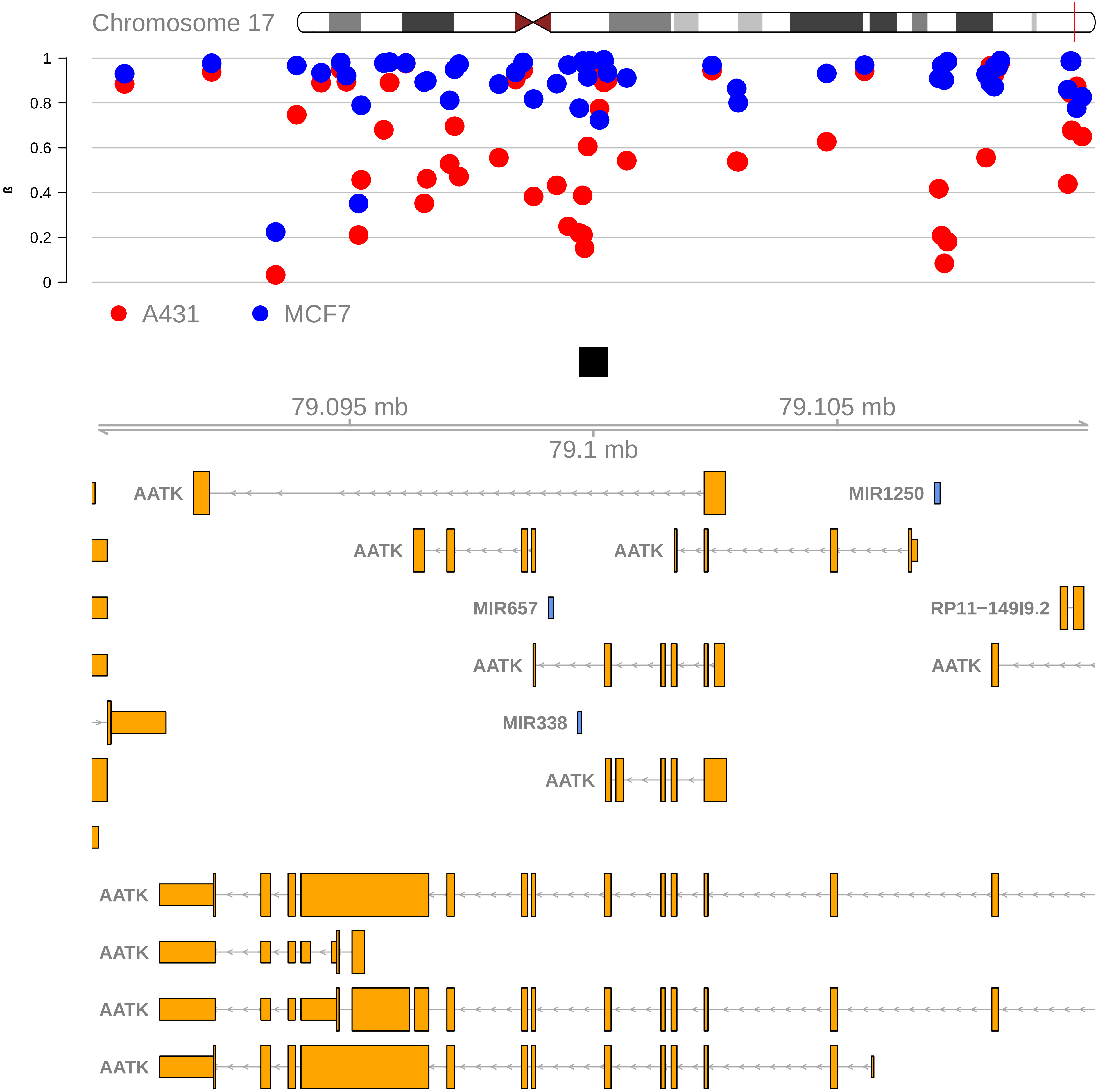
Chromosome 17
1
0.8
0.6
0.4
0.2
0
ß
A431
MCF7
79.095 mb
79.105 mb
79.1 mb
AATK
MIR1250
AATK
AATK
MIR657
RP11−149I9.2
AATK
AATK
MIR338
AATK
AATK
AATK
AATK
AATK
